# Reliability of Sleep Measures from Four Personal Health Monitoring Devices Compared to Research-Based Actigraphy and Polysomnography

**DOI:** 10.3390/s16050646

**Published:** 2016-05-05

**Authors:** Janna Mantua, Nickolas Gravel, Rebecca M. C. Spencer

**Affiliations:** 1 Neuroscience & Behavior Program, University of Massachusetts, Amherst, 135 Hicks Way, Amherst, MA 01003, USA; jmantua@cns.umass.edu; 2 Department of Psychological & Brain Sciences, University of Massachusetts, Amherst, 135 Hicks Way, Amherst, MA 01003, USA; ngravel007@gmail.com

**Keywords:** actigraphy, wearables, validation, polysomnography, measurement

## Abstract

Polysomnography (PSG) is the “gold standard” for monitoring sleep. Alternatives to PSG are of interest for clinical, research, and personal use. Wrist-worn actigraph devices have been utilized in research settings for measures of sleep for over two decades. Whether sleep measures from commercially available devices are similarly valid is unknown. We sought to determine the validity of five wearable devices: Basis Health Tracker, Misfit Shine, Fitbit Flex, Withings Pulse O2, and a research-based actigraph, Actiwatch Spectrum. We used Wilcoxon Signed Rank tests to assess differences between devices relative to PSG and correlational analysis to assess the strength of the relationship. Data loss was greatest for Fitbit and Misfit. For all devices, we found no difference and strong correlation of total sleep time with PSG. Sleep efficiency differed from PSG for Withings, Misfit, Fitbit, and Basis, while Actiwatch mean values did not differ from that of PSG. Only mean values of sleep efficiency (time asleep/time in bed) from Actiwatch correlated with PSG, yet this correlation was weak. Light sleep time differed from PSG (nREM1 + nREM2) for all devices. Measures of Deep sleep time did not differ from PSG (SWS + REM) for Basis. These results reveal the current strengths and limitations in sleep estimates produced by personal health monitoring devices and point to a need for future development.

## 1. Introduction

Personal health monitoring devices have rapidly gained popularity in the general public, providing the potential to improve feedback and motivation [1,2] towards improvements in physical activity, dietary intake, and sleep. These commercially available products have begun to overcome limitations in cost (as low as $60) and usability, and have been validated several times in the area of physical activity [3,4,5,6]. Yet whether devices are sufficiently accurate in the sleep domain is not well understood. 

Polysomnography (PSG) has long been the “gold standard” for measuring sleep. PSG provides general sleep measures, such as total sleep time (TST) and sleep efficiency (SE), in addition to providing measures of specific sleep stages. However, despite clear benefits, PSG is costly, arduous to apply, and can be intrusive to sleep itself, making the search for alternatives essential to the field [7]. 

Actigraphy has been validated for general measures of sleep (e.g., [8]) and has proven valuable for clinical and research use as it is relatively inexpensive, non-intrusive, and does not require a sleep technician for application. However, a limitation of the validated research-based actigraphy is that it relies on hand-scored data using participant diaries of events (bedtime/wake time and time in bed; [8,9]). Moreover, reliability of sleep staging generated by commercially available devices has received little attention. 

Here we sought to determine the validity of sleep measures—TST, SE, light sleep time, and deep sleep time—from four commercially-available personal health monitoring devices compared to PSG. For direct comparison to these devices, we also assessed the validity of TST and SE measures from a research-validated actigraph compared to PSG. Such comparisons are essential for understanding the role of these measures for personal health monitoring and for clinical consideration.

## 2. Materials

### 2.1. Actigraphy

The Basis Health Tracker (2014 edition; Intel Corp, Santa Clara, CA, USA) is a wristwatch with an embedded actigraph and automatic sleep detection. Data were uploaded to the user website which generated measures of sleep (see Table 1).

The Fitbit Flex (Fitbit Inc., San Francisco, CA, USA) is a wristband with an embedded actigraph. Sleep-tracking mode is initiated by repeatedly tapping on the band for 1–2 s until two dimming lights appear on the device’s display. The same tapping pattern is used to stop sleep-tracking, at which point the display flashes five LEDs to signal “wake mode.” Data from the Fitbit were uploaded to the user website which generated sleep measures.

The Misfit Shine (Misfit Wearables, San Francisco, CA, USA) was worn with the provided wrist strap. Sleep-tracking mode was set to automatic. Data were uploaded to the device’s mobile application via Bluetooth and measures were extracted from the application. 

The Withings Pulse O2 (Withings, Issy-les-Moulineaux, France) was worn on the wrist with the supplied wrist strap. Sleep tracking is manually activated by swiping the finger across the face of the device and deactivated in the same way. Recorded data were uploaded via Bluetooth to the mobile application. 

The Actiwatch Spectrum (Philips Respironics, Bend, OR, USA) is a wristwatch with embedded accelerometer and off-wrist detection. The Actiwatch was set to record the mean activity in 15-s epochs. Participants were instructed to press an event-marker button to denote bedtime and wake time. 

### 2.2. Polysomnography

Polysomnography was recorded with an Aura PSG ambulatory system (Natus Neurology, West Warwick, RI, USA). The montage included six EEG leads (O1, O2, C3, C4, F3, F4, Cz), two EOG leads (one on the side of each eye), two chin EMG leads, two mastoid electrodes, and one ground electrode on the forehead.

## 3. Methods

### 3.1. Participants

Participants were 40 healthy young adults (19 female) aged 18–30 years (mean age = 22.37 years; SD = 4.92). To be eligible, participants were required to have no history of sleep or neurological disorder. Participants were compensated monetarily. Procedures were approved by the University of Massachusetts, Amherst Institutional Review Board (IRB). All participants provided written informed consent prior to participation. 

### 3.2. Procedure

Data were collected as part of a larger study aimed at algorithm development for a novel wearable device (*i.e.*, a device worn on the upper arm; data from this device is not presented). Importantly, the parent companies of the reported devices provided no funding for this study. Moreover, the sponsor nor any other entity contributed to the present analyses or data presentation.

Prior to testing, all devices were time-synchronized and configured on the same computer. Sleep was recorded in the participant’s home. PSG was applied and the participant was fitted with the five devices 1–2 h prior to their normal bedtime. The Basis and the Misfit were applied to one wrist while the Fitbit, Withings, and Actiwatch were placed on the opposite wrist. Wrist placement (right/left) was counterbalanced across participants. Subsequently, participants were verbally instructed and left with printed instructions as to how to properly initiate the devices at bedtime (Fitbit, Withings, and Actiwatch). The following morning, participants removed the devices and completed a sleep diary, noting time in bed and wake time.

### 3.3. Analysis

Two trained sleep researchers scored all PSG records according to criteria outlined in the American Academy of Sleep Medicine (AASM; [10]). Sleep onset latency (Table 1) was determined by comparing participant self-reported sleep time (via sleep diary) with sleep onset determined by PSG. Actiwatch data was downloaded and analyzed with the provided software (Actiware v.6). Actiwatch data were scored for sleep or wake in 15-s epochs using the Actiware software (Philips Respironics, Bend, OR, USA) default algorithm. Sleep was identified through a combination of sleep diaries and event markers. 

There were three phases of analysis: (1) a qualitative examination of device success or failure, (2) measurements of validity for all devices (“unadjusted analyses”); and (3) measurements of validity for successful devices (“adjusted analyses”). For the second and third phases, data were analyzed using SPSS version 21. Bland-Altman plots were used to evaluate the agreement between PSG and device measures. A Wilcoxon Signed-Rank (WSR) test was used to determine whether differences between devices and PSG were significant (*i.e.*, significance indicates values are from two distinct sources). Pearson’s correlations were used to assess concordance between sleep parameters from devices and PSG. Measures adopted from each device are defined in Table 1.

## 4. Results

### 4.1. Device Success and Failure

Device failures are presented in Table 2. Several devices exhibited gross mis-estimation, which we quantified as TST > 2 h over PSG TST (1 Actiwatch, 1 Basis, 1 Fitbit, 8 Misfit, 1 Withings). Participants also failed to initiate “sleep mode” in the devices requiring such (*i.e.*, Fitbit and Withings). The outcome was poorest for the Fitbit: 9 devices were not initiated, relative to 3 devices for Withings. There were also a number of other miscellaneous device malfunctions that were due to hardware/software error rather than human error. These issues included an inability of the software to detect any data or an inability of the device to make a connection with the software (*i.e.*, preventing download of data: 3 Basis, 7 Misfit, 4 Actiwatch). When examining data loss as a whole, the failure rates of the Misfit and Fitbit were the most pronounced. In the unadjusted analyses, all data points, including those with gross mis-estimation, were included. On the other hand, only “successful” devices were analyzed in adjusted analyses to determine whether device data are valid when the “failure” points are overcome. 

### 4.2. Sleep Characteristics

Despite sleeping with multiple devices, participant sleep was in the range of normal (e.g., [11]). Characteristics of participant sleep are presented in Table 3.

### 4.3. Total Sleep Time

**Figure 1:**
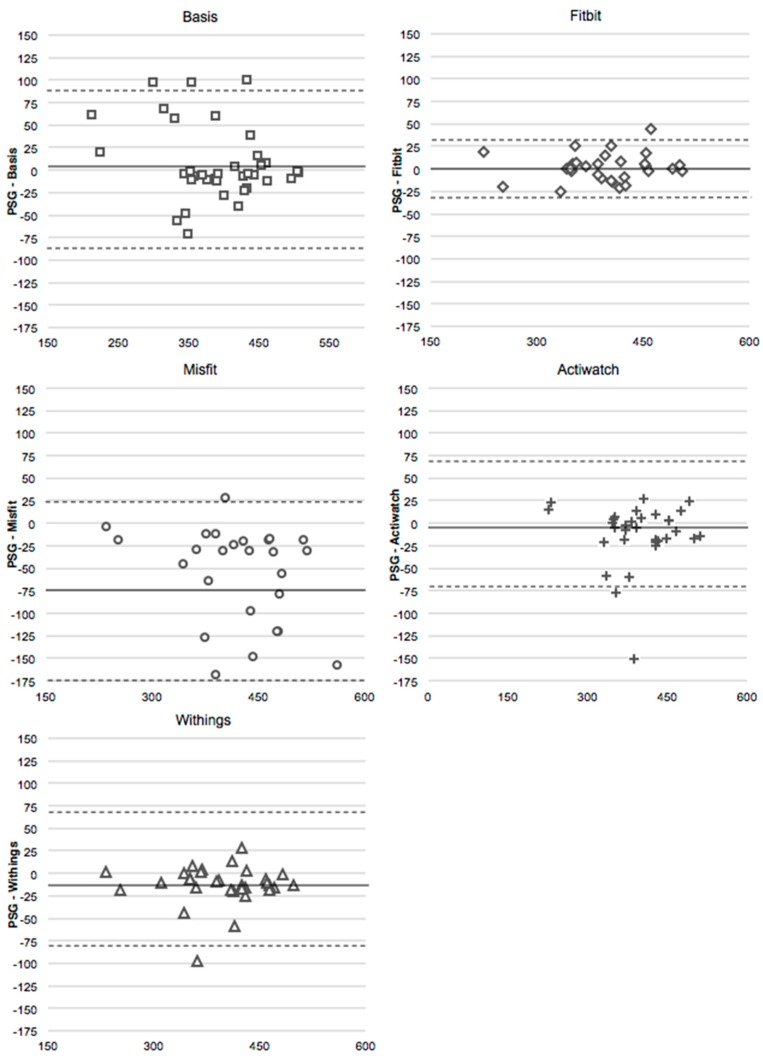
Bland-Altmann plots for total sleep time (minutes) for the Basis, the Fitbit, the Misfit, the Spectrum, and the Withings compared to PSG (adjusted values). The x-axis is the mean of the two devices, and the y-axis represents PSG minus the device. Dotted line represents two standard deviations from the mean.

For both the unadjusted and adjusted analyses, WSR tests indicated that TST for all devices did not differ from the measures of TST from PSG (Table 4). The Misfit neared significance as it tended to overestimate TST relative to PSG (Figure 1). TST recorded by all devices significantly and strongly correlated with that of PSG (Table 5). It is notable that correlations between devices and PSG were strongest for devices requiring manual sleep activation (*i.e.*, Actiwatch, Fitbit, and Withings).

### 4.4. Sleep Efficiency

**Figure 2:**
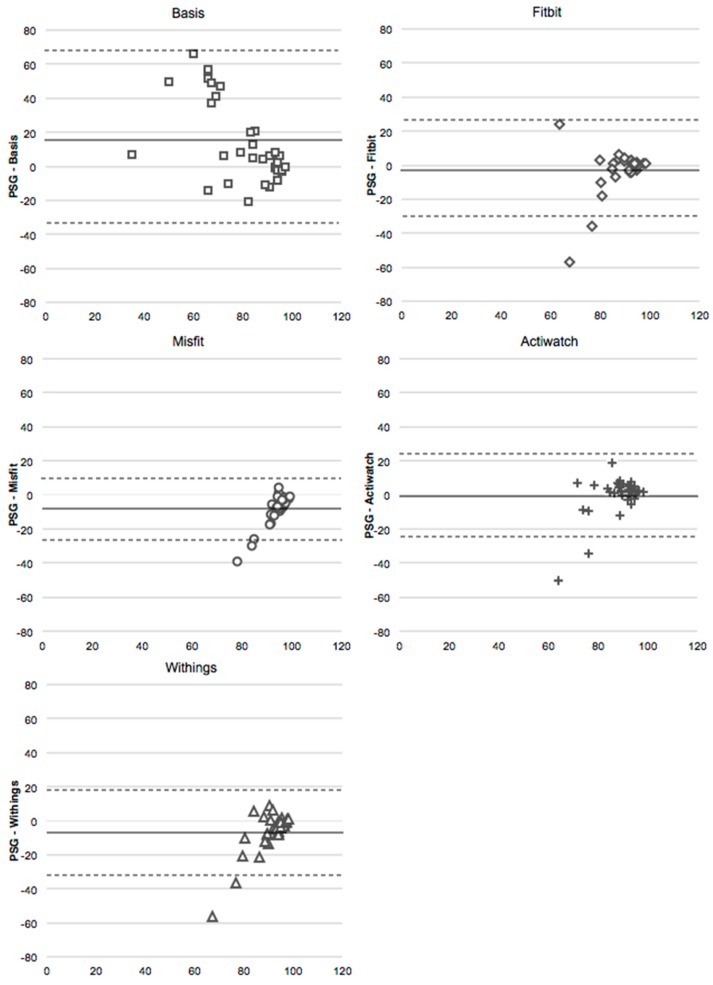
Bland-Altmann plots for sleep efficiency (%) for the Basis, Fitbit, Misfit, Withings, and Actiwatch compared to PSG (adjusted values). The x-axis is the mean of the two devices, and the y-axis represents PSG minus the device. Dotted line represents two standard deviations from the mean.

Based on both the unadjusted and adjusted analyses, Actiwatch and Fitbit, measures of SE were not different from PSG based on WSR tests. On the other hand, in both sets of analyses, the Misfit and Withings overestimated SE relative to PSG, and the Basis underestimated SE (Figure 2). In the unadjusted analyses, no devices correlated with PSG, although there was a trend for a weak correlation between the Actiwatch and PSG (p = 0.07; Table 5). In the adjusted analyses, SE from the Actiwatch weakly correlated with that of PSG, while SE of Fitbit, Misfit, Basis and Withings did not.

### 4.5. Deep Sleep

**Figure 3:**
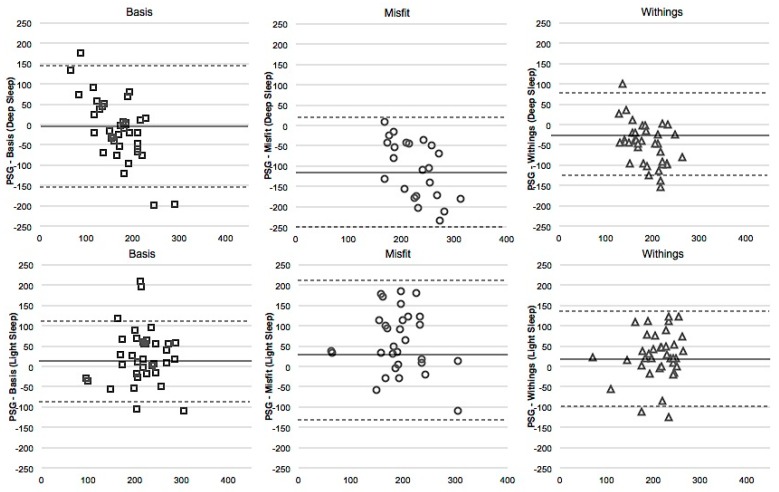
Bland-Altmann plots for Deep Sleep (**top row**) and Light Sleep (**bottom row**), each in minutes, for the Basis, the Misfit, and the Withings (adjusted values). The x-axis is the mean of the two devices, and the y-axis represents PSG minus the device. Dotted line represents two standard deviations from the mean.

WSR tests and the Bland-Altman plots suggest similar results between unadjusted and adjusted data. In both unadjusted and adjusted analyses, measures of Deep sleep are distinct from PSG for Misfit and Withings. These devices tended to overestimate Deep sleep (Figure 3, top row). The Basis underestimated Deep sleep when Deep sleep was low and overestimated Deep sleep when it was high. Unadjusted, Withings significantly but weakly correlated with PSG (Table 5), and the Basis correlated with PSG marginally and weakly (p = 0.09). However, in the adjusted analyses, only the Withings weakly correlated with PSG.

### 4.6. Light Sleep

For both the unadjusted and adjusted analyses, WSR tests and Bland-Altman plots revealed a significant distinction between Light sleep measures from all devices compared to PSG (Figure 3, bottom row). All devices tended to underestimate Light sleep. In unadjusted correlations, Withings’ measure of Deep sleep weakly correlated significantly with PSG, while there was a weak trend between PSG and Misfit (p = 0.09), and between PSG and Basis (p = 0.08; Table 5). In the adjusted analyses, the Withings and Misfit were moderately correlated with PSG.

## 5. Discussion

This study was designed to validate and compare sleep measures (TST, SE, Light sleep, and Deep sleep) recorded from several commercially available, wrist-worn personal health monitoring devices against PSG, the “gold standard” for sleep monitoring. Overall, we found specific categories of device data did not differ from PSG measures (summarized in Table 6), yet many devices provided unusable data.

With and without the exclusion of data points exhibiting gross mis-estimation, TST measures from all devices were did not differ from PSG measures. On the other hand, only the Actiwatch and Fitbit devices provided SE measures that did not differ from PSG measured SE (via WSR). Further, only the Actiwatch correlated with PSG for SE, albeit weakly. The Basis, Misfit, and Withings reported Light and Deep sleep measures. However, Light sleep measures were considered distinct from that of PSG based on WSR tests, and only estimates of Light sleep from the Misfit and Withings moderately correlated with PSG measures of light sleep (nREM1 + nREM2). The Basis measure of Deep sleep was not different from that of PSG, and the Withings estimate of Deep sleep was the only to correlate with PSG (SWS + REM). Thus, several devices were able to accurately assess TST and SE, yet no devices provided reliable staging data.

The high reliability of the Actiwatch Spectrum for estimating TST is consistent with other studies [12]. Based on such evidence, the Actiwatch is widely used both in research (e.g., [13,14] and clinical (see [15]) settings. However, to achieve this reliability, participants must note bed and wake times in a sleep diary and trained researchers must score the data, which is labor intensive and fails to provide real-time feedback. In the absence of this, the Fitbit and Withings obtained TST measures that did not differ significantly from that of PSG, contrary to others’ findings ([16,17,18]). The Fitbit and Basis also had SE measures that did not differ from PSG and were within the same narrow range of bias as the Actiwatch, yet correlations with PSG were not significant for these devices. Therefore, the recommendation of these devices for research purposes may be premature. 

Two devices, the Fitbit and the Withings, required user input to initiate “sleep mode,” which may both impede and facilitate data validity. For example, for the Fitbit, measures of SE and TST did not differ significantly from PSG. However, sleep mode initiation may have been an obstacle to measurement, as Fitbit data for 9 participants were lost. These findings are consistent with a recent longitudinal investigation that identified very high data loss for this device [19]. Yet it is important to note that user-input *per se* was not the issue, as the Withings exhibited fewer lost data points than the Fitbit (3 as opposed to 9). We presume this difference occurred because the Withings device clearly confirms the user is in sleep mode (*i.e.*, “Goodnight” flashing on the screen), whereas the Fitbit notifies the user of sleep mode less clearly (*i.e.*, a series of vibrations and lights signals both “sleep mode” and “wake mode”). Therefore, the mode of input for the Fitbit could induce data loss, which minimizes reliability of the device. Devices that did not require user input, the Basis and the Misfit (as we used the automatic mode in this study), performed less satisfactory. Both the Basis and Misfit were not unreliable in their measure of TST, however the range of bias was large (approximately ±75 min). The Basis was similarly variable for measures of SE relative to PSG.

A unique aspect of this study was the assessment of the validity of measures of Light and Deep sleep, a popular output feature of commercially available devices. Light and Deep sleep measures from all tested devices, the Misfit, Basis, and Withings, were not comparable to PSG. Notably, both Light and Deep sleep measures from the Withings correlated with the respective measures from PSG. However, Light sleep was underestimated and Deep sleep was significantly overestimated. The Misfit measure of Light sleep weakly correlated with that from PSG however it was significantly underestimated by over an hour (average bias of 79 min) while Deep sleep was significantly overestimated to an even greater extent (average bias of 107 min). Measures of Light and Deep sleep from the Basis showed little evidence of reliability. 

Reliability of Light and Deep sleep measures was based on the assumption that Light sleep refers to nREM1 and nREM2 and Deep sleep refers to SWS and REM. Light sleep is typically defined as the combination of nREM1 and nREM2 in the sleep literature (e.g., [20,21,22]). However, Deep sleep is typically defined as nREM3 and nREM4 (*i.e.*, SWS) with REM a distinct measure. Algorithmically, devices are likely to collapse SWS and REM, as these stages are similarly characterized by low movement. This assumption is supported by the available literature on similar devices (e.g., [23]). Nonetheless, companies making these devices may have a different underlying assumption of what Light and Deep sleep measures capture compared to PSG.

In addition to our assumptions regarding Light and Deep sleep, there are other possible limitations to this study. First, there was little variation in the measure of SE across the young adult population studied here which may explain the lack of correlation between Basis, Misfit, Fitbit, and Withings measures of SE with PSG measured SE. Second, we excluded data for devices that recorded a TST >2 h from the TST from PSG (discussed further below). Given that there is no clear way to identify devices with gross failure, we chose a threshold cutoff that could be consistently applied across all devices. Most commonly excluded was data from the Misfit, which may have resulted in overestimation of the strength of this device. Third, most devices generate global measures and epoch-by-epoch data was not available particularly at the appropriate temporal resolution. As such, we could not conduct epoch-by-epoch analyses of sensitivity and specificity (see [24]).

We chose to perform comparative analyses despite high data loss. In the adjusted analyses, we also chose to exclude data points deemed as a gross failure based on TST, the measure found to be most reliable across devices. We were interested in comparing devices without the failed devices given that we speculate instances of gross failure are indistinguishable from other device errors. For example, use of the Misfit device resulted in high data loss due to mis-estimation. Although the cause of these errors is unknown, they may be either human-related (e.g., the watch was not tight enough to the wrist) or hardware-related (e.g., a glitch in the recording system), and thus these errors were grouped with more blatant data loss instances (e.g., user-initiation errors). Although the removal of such data points may bias results in favor of the devices, we thought it important to examine data validity when “failure” points are overcome. In that way, the true validity of the devices could be determined. Nevertheless, comparative analyses should be interpreted with the knowledge that errors are a frequent impediment to data acquisition. 

Collectively, these data suggest that the value of commercially available devices for measurement of sleep depends on the measure of interest and application. Total sleep time, and in some cases, sleep efficiency, can be monitored by wrist-worn, commercially available devices, yet the reliability of these devices remains low. These devices do not yet yield sufficient information for accurate sleep staging, even on a superficial level (e.g., Light *vs.* Deep). Therefore, research focusing on habitual total sleep time could utilize some of these devices, while work focusing on sleep efficiency or staging, as well as clinical applications such as detection of apneic events, should continue to rely on PSG. Given the continuing advancement of sleep-detecting algorithms and measurement techniques, it is not unrealistic to believe that more complete and commercially available sleep monitoring systems will be available in the near future. 

## Figures and Tables

**Table 1:** Measures compared across devices.

	TST	SE	Light	Deep
Polysomnography	Time in bed–time awake	TST/time in bed	nREM1 + nREM2	SWS + REM
Actiwatch	Time in bed–time awake	TST/time in bed	-	-
Basis	Asleep	Sleep Score	Light	Deep + REM
Fitbit	Actual Sleep Time	Actual Sleep Time/You Were in Bed for	-	-
Misfit	Light + Restful	Light + Restful/Light + Restful + Awake	Light	Restful
Withings	Sleep Duration	Sleep Duration/In Bed Duration	Light	Deep

**Table 2:** Reasons for Device Failure.

	Gross Mis-Estimation	Miscellaneous	User Error
Actiwatch	1	4	-
Basis	1	3	-
Fitbit	1	0	9
Misfit	8	7	-
Withings	1	0	3

**Table 3:** Sleep characteristics based on polysomnography.

Sleep Parameter	Mean ± SD
Time in bed (min)	465.98 ± 96.87
Total sleep time (min)	397.44 ± 63.63
SE (%)	84.61 ± 18.15
WASO (min)	32.22 ± 43.60
Sleep onset latency (min)	35.09 ± 67.78
nREM1 (%)	9.58 ± 3.64
nREM2 (%)	47.79 ± 8.20
SWS (%)	23.20 ± 7.51
REM (%)	19.43 ± 5.03

**Table 4:** Z-values of Wilcoxon Signed Rank Test. Bold indicates a significant difference between devices (p < 0.05).

	TST		SE		Light		Deep	
	Unadj.	Adj.	Unadj.	Adj.	Unadj.	Adj.	Unadj.	Adj.
Actiwatch	–1.62	−1.40	−1.62	−1.42	-	-	-	-
Basis	−1.41	−1.41	**−2.56**	**−2.37**	**−2.22**	**−2.22**	−1.21	−1.51
Fitbit	−0.37	−0.37	−0.68	−0.68	-	-	-	-
Misfit	**−4.21**	**−3.62**	**−4.01**	**−3.90**	**−4.04**	**−3.58**	**−4.68**	**−4.35**
Withings	**−3.84**	**−3.76**	**−3.49**	**−3.84**	**−3.21**	**−2.89**	**−4.23**	**−4.25**

**Table 5:** Correlations of measures (r) from each device with PSG. Bold indicates significance (p < 0.05) and ^#^ indicates marginal significance (p < 0.1).

	TST		SE		Light		Deep	
	Unadj.	Adj.	Unadj.	Adj.	Unadj.	Adj.	Unadj.	Adj.
Actiwatch	**0.87**	**0.94**	0.30 ^#^	**0.35**	-	-	-	-
Basis	**0.84**	**0.84**	0.26	0.28	0.30 ^#^	0.30 ^#^	0.27	0.28 ^#^
Fitbit	**0.97**	**0.97**	0.21	0.21	-	-	-	-
Misfit	**0.76**	**0.87**	−0.20	−0.07	0.31 ^#^	**0.40**	0.20	0.19
Withings	**0.84**	**0.94**	0.17	0.21	**0.34**	**0.39**	**0.36**	**0.36**

**Table 6:** Percentage error (average and maximum) for each device. Each value is calculated as 100 * [Absolute value of (Parameter_device_ − Parameter_PSG_)/Parameter_PSG_.]. Numbers represent unadjusted values.

	TST		SE		Light		Deep	
	Avg.	Max.	Avg.	Max.	Avg.	Max.	Avg.	Max.
Actiwatch	5.82	48.31	10.37	128.21	-	-	-	-
Basis	7.82	28.33	22.22	69.89	23.90	68.97	35.02	133.11
Fitbit	2.97	8.23	11.57	146.15	-	-	-	-
Misfit	15.26	54.90	13.83	89.90	33.82	72.66	69.58	155.94
Withings	6.00	57.30	11.43	143.59	24.06	93.50	37.202	107.75
